# 2-Di­phenyl­phosphanyl-1-methyl-1*H*-benzimidazole

**DOI:** 10.1107/S1600536813020588

**Published:** 2013-07-31

**Authors:** Darkus E. Jenkins, Zerihun Assefa

**Affiliations:** a North Carolina A&T State University, 1601 E Market St., Department of Chemistry, Greensboro, NC 27411, USA

## Abstract

In the title compound, C_20_H_18_N_2_P, the P atom is bonded to the two phenyl and imidazole groups, with an average P—C bond length of 1.828 (2) Å. The three C—P—C bond angles have values consistent with a tetra­hedral geometry around the P atom with the fourth site occupied by a H atom. Crystal packing is through van der Waals inter­actions.

## Related literature

For the first synthesis of the title compound and related systems, see: Moore & Whitesides (1982 ▶). For multimode coordination of di­phenyl­phosphine-substituted benzimidazoles featuring ethyl­ene linkers, see: Hahn *et al.* (2010 ▶). For amino-group linkers, see: Braunstein *et al.* (1997 ▶). For the coordination of the N,P-type ligand (1-benzyl-2-imidazol­yl)di­phenyl­phos­phine (BzimPh_2_P) with several metal ions, see: Burini *et al.* (2000 ▶). For silver complexes with the same ligand, see: Bachechi *et al.* (2001 ▶). 
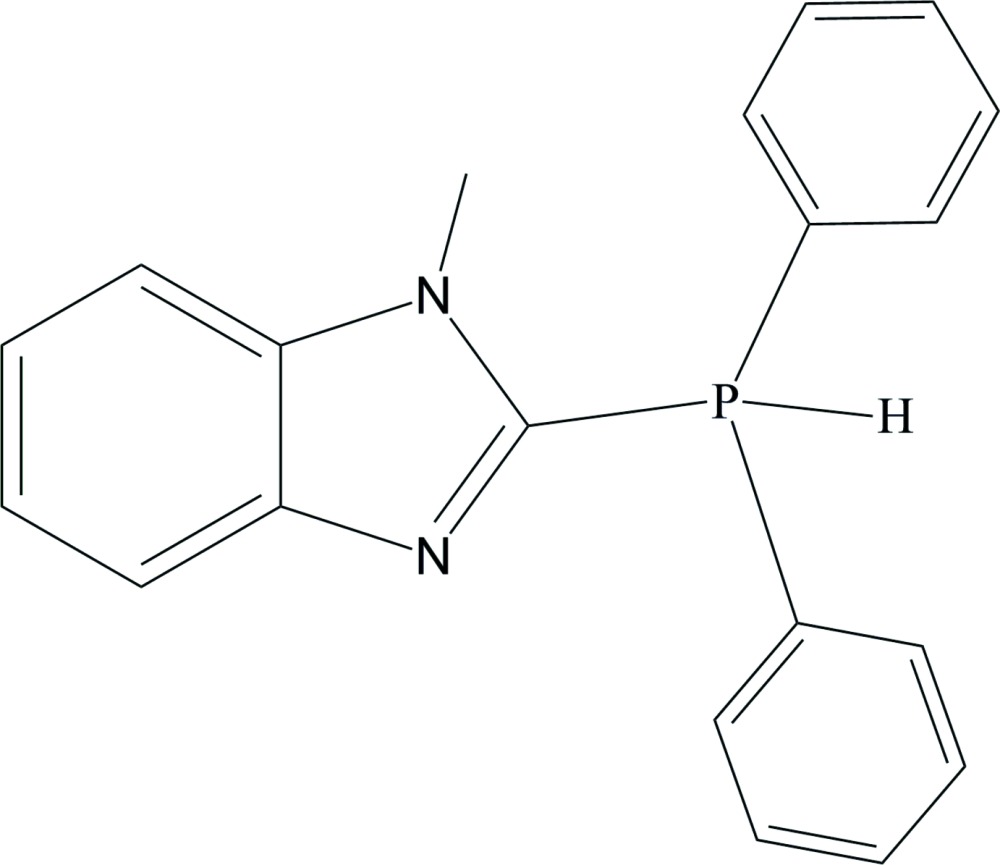


## Experimental

### 

#### Crystal data

C_20_H_18_N_2_P
*M* *_r_* = 317.33
Triclinic, 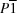

*a* = 9.574 (2) Å
*b* = 9.904 (3) Å
*c* = 10.513 (3) Å
α = 74.215 (7)°
β = 67.172 (7)°
γ = 70.346 (7)°
*V* = 853.6 (4) Å^3^
*Z* = 2
Mo *K*α radiation
μ = 0.16 mm^−1^
*T* = 200 K
0.50 × 0.50 × 0.05 mm

#### Data collection

Bruker SMART X2S diffractometer
Absorption correction: multi-scan (*SADABS*; Bruker, 2008 ▶) *T*_min_ = 0.924, *T*_max_ = 0.992
8036 measured reflections
2973 independent reflections
2497 reflections with *I* > 2σ(*I*)
*R*_int_ = 0.027

#### Refinement

*R*[*F* ^2^ > 2σ(*F* ^2^)] = 0.040
*wR*(*F* ^2^) = 0.118
*S* = 1.06
2973 reflections
212 parameters
H atoms treated by a mixture of independent and constrained refinement
Δρ_max_ = 0.30 e Å^−3^
Δρ_min_ = −0.18 e Å^−3^

### 

Data collection: *SMART* (Bruker, 2008 ▶); cell refinement: *SAINT* (Bruker, 2008 ▶); data reduction: *SAINT*; program(s) used to solve structure: *SHELXS97* (Sheldrick, 2008 ▶); program(s) used to refine structure: *SHELXL97* (Sheldrick, 2008 ▶); molecular graphics: *JMol* (Hanson, 2010 ▶); software used to prepare material for publication: *publCIF* (Westrip, 2010 ▶).

## Supplementary Material

Crystal structure: contains datablock(s) I, New_Global_Publ_Block. DOI: 10.1107/S1600536813020588/kp2457sup1.cif

Structure factors: contains datablock(s) I. DOI: 10.1107/S1600536813020588/kp2457Isup2.hkl

Supplementary material file. DOI: 10.1107/S1600536813020588/kp2457Isup5.cml

Additional supplementary materials:  crystallographic information; 3D view; checkCIF report

## Figures and Tables

**Fig. 1.**
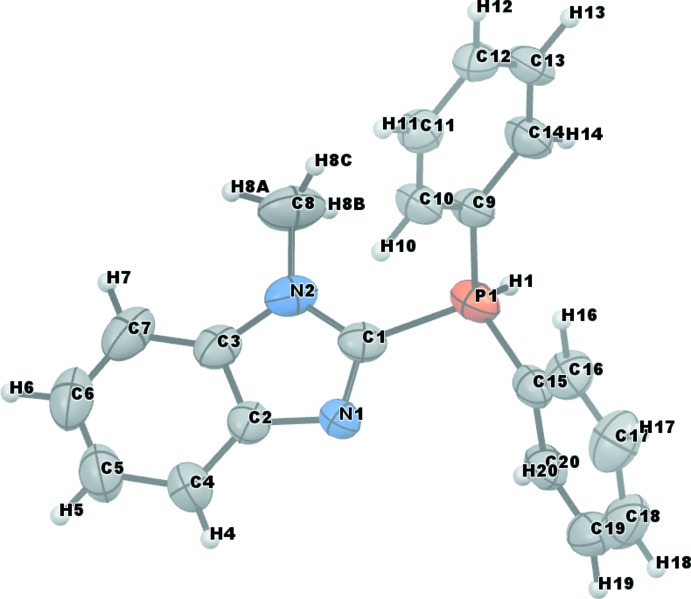
The molecular structure of the compound. Thermal ellipsoids for non- hydrogen atoms are drawn at 50% probability level.

## supplementary crystallographic information

### Comment

Over the past four decades attention has been given to bi-functional ligands with regards to bringing two metal atoms in close proximity. In particular, interest has been given to the N, P-type ligands. These ligands follow the Lewis soft acid/base chemistry when coordinating with group 11 metals. Although N is to some extent a lesser soft base than P, (N is a stronger σ-donor and poorer π-acceptor than P) its ability to bind with the softer group 11 metals such as gold (I) is well documented. This multi-mode coordination has proven to bring homo- and heteronuclear atoms together with short distances. These ligands are thought to offer stability for metal – metal interactions. Diphenylphosphine-substituted benzimidazoles featuring ethylene or methylene linkers between the benzimidazole and the phosphine groups have been studied in few instances by Hahn *et al.* (2010) as are with amino linkers by Braunstein *et al.* (1997). Burini *et al.* (2000) have extensively studied the N, P type ligand, (1-benzyl-2-imidazolyl)diphenylphosphine (BzimPh2P) to understand the behaviour of coordination with various metal systems. The coordination of the ligand with gold (I), silver (I), copper (I), rhodium (I), iridium (I), mercury (II), zinc (II), and cadmium (II) metal ions has been studied utilizing the monodentate and bidentate features of the ligand. Through continued work Bachechi *et al.* (2001) have studied the X-ray crystal structures of the silver complexes with the same ligand. Although the synthesis of the title compound has been reported by Moore *et al.* (1982) surprisingly there has been no structural and coordination work of the this potentially bidentate system.

### Experimental

The compound was synthesized by reacting 1-methylbenzimidazole with chlorodiphenylphosphine in the presence of n-BuLi at 195 K. Single crystals were obtained from warm hexanes solution.

### Crystal data

**Table:**

C_20_H_18_N_2_P	*Z* = 2
*M**_r_* = 317.33	*F*(000) = 334
Triclinic, *P*1	*D*_x_ = 1.235 Mg m^−^^3^
Hall symbol: -P 1	Mo *K*α radiation, λ = 0.71073 Å
*a* = 9.574 (2) Å	Cell parameters from 3787 reflections
*b* = 9.904 (3) Å	θ = 2.2–24.0°
*c* = 10.513 (3) Å	µ = 0.16 mm^−^^1^
α = 74.215 (7)°	*T* = 200 K
β = 67.172 (7)°	Plate, colourless
γ = 70.346 (7)°	0.50 × 0.50 × 0.05 mm
*V* = 853.6 (4) Å^3^	

### Data collection

**Table:**

Bruker SMART X2S diffractometer	2973 independent reflections
Radiation source: fine-focus sealed tube	2497 reflections with *I* > 2σ(*I*)
Graphite monochromator	*R*_int_ = 0.027
automatic scans	θ_max_ = 25.1°, θ_min_ = 2.1°
Absorption correction: multi-scan (*SADABS*; Bruker, 2008)	*h* = −11→11
*T*_min_ = 0.924, *T*_max_ = 0.992	*k* = −11→11
8036 measured reflections	*l* = −12→12

### Refinement

**Table:**

Refinement on *F*^2^	Primary atom site location: structure-invariant direct methods
Least-squares matrix: full	Secondary atom site location: difference Fourier map
*R*[*F*^2^ > 2σ(*F*^2^)] = 0.040	Hydrogen site location: inferred from neighbouring sites
*wR*(*F*^2^) = 0.118	H atoms treated by a mixture of independent and constrained refinement
*S* = 1.06	*w* = 1/[σ^2^(*F*_o_^2^) + (0.0576*P*)^2^ + 0.2237*P*] where *P* = (*F*_o_^2^ + 2*F*_c_^2^)/3
2973 reflections	(Δ/σ)_max_ < 0.001
212 parameters	Δρ_max_ = 0.30 e Å^−^^3^
0 restraints	Δρ_min_ = −0.18 e Å^−^^3^

### Special details

**Table:**

Geometry. All e.s.d.'s (except the e.s.d. in the dihedral angle between two l.s. planes) are estimated using the full covariance matrix. The cell e.s.d.'s are taken into account individually in the estimation of e.s.d.'s in distances, angles and torsion angles; correlations between e.s.d.'s in cell parameters are only used when they are defined by crystal symmetry. An approximate (isotropic) treatment of cell e.s.d.'s is used for estimating e.s.d.'s involving l.s. planes.
Refinement. Refinement of *F*^2^ against ALL reflections. The weighted *R*-factor *wR* and goodness of fit *S* are based on *F*^2^, conventional *R*-factors *R* are based on *F*, with *F* set to zero for negative *F*^2^. The threshold expression of *F*^2^ > σ(*F*^2^) is used only for calculating *R*-factors(gt) *etc*. and is not relevant to the choice of reflections for refinement. *R*-factors based on *F*^2^ are statistically about twice as large as those based on *F*, and *R*- factors based on ALL data will be even larger.

### Fractional atomic coordinates and isotropic or equivalent isotropic displacement parameters (Å^2^)

**Table:**

	*x*	*y*	*z*	*U*_iso_*/*U*_eq_	
P1	0.78385 (6)	0.78051 (5)	0.17374 (6)	0.05124 (19)	
N1	0.70104 (17)	0.53236 (16)	0.18024 (16)	0.0448 (4)	
N2	0.73242 (17)	0.53550 (18)	0.37950 (16)	0.0511 (4)	
C1	0.73869 (19)	0.6038 (2)	0.24516 (19)	0.0449 (4)	
C2	0.6711 (2)	0.4084 (2)	0.27610 (19)	0.0461 (4)	
C3	0.6890 (2)	0.4095 (2)	0.4008 (2)	0.0489 (5)	
C4	0.6339 (3)	0.2909 (2)	0.2604 (2)	0.0617 (5)	
H4	0.6225	0.2878	0.1755	0.074*	
C5	0.6143 (3)	0.1788 (3)	0.3722 (3)	0.0731 (6)	
H5	0.5911	0.0965	0.3631	0.088*	
C6	0.6276 (3)	0.1836 (3)	0.4966 (3)	0.0754 (7)	
H6	0.6099	0.1060	0.5721	0.090*	
C7	0.6658 (3)	0.2977 (3)	0.5147 (2)	0.0668 (6)	
H7	0.6759	0.3001	0.6003	0.080*	
C9	0.9911 (2)	0.73075 (19)	0.1591 (2)	0.0454 (4)	
C10	1.0919 (2)	0.5953 (2)	0.1370 (2)	0.0503 (5)	
H10	1.0539	0.5222	0.1285	0.060*	
C11	1.2477 (2)	0.5647 (2)	0.1273 (2)	0.0516 (5)	
H11	1.3154	0.4708	0.1129	0.062*	
C12	1.3044 (2)	0.6700 (2)	0.1383 (2)	0.0513 (5)	
H12	1.4110	0.6491	0.1320	0.062*	
C13	1.2059 (2)	0.8058 (2)	0.1585 (2)	0.0613 (6)	
H13	1.2453	0.8791	0.1646	0.074*	
C14	1.0496 (2)	0.8365 (2)	0.1700 (2)	0.0568 (5)	
H14	0.9821	0.9302	0.1855	0.068*	
C15	0.7894 (2)	0.79884 (18)	−0.0053 (2)	0.0468 (4)	
C16	0.9256 (2)	0.7847 (2)	−0.1200 (2)	0.0578 (5)	
H16	1.0245	0.7596	−0.1067	0.069*	
C17	0.9192 (3)	0.8067 (2)	−0.2525 (3)	0.0740 (7)	
H17	1.0133	0.7977	−0.3303	0.089*	
C18	0.7758 (3)	0.8418 (2)	−0.2731 (3)	0.0763 (7)	
H18	0.7716	0.8549	−0.3646	0.092*	
C19	0.6397 (3)	0.8576 (2)	−0.1605 (3)	0.0718 (7)	
H19	0.5410	0.8827	−0.1742	0.086*	
C20	0.6462 (2)	0.8373 (2)	−0.0282 (2)	0.0578 (5)	
H20	0.5516	0.8497	0.0488	0.069*	
H1	0.6977 (18)	0.8932 (17)	0.2303 (16)	0.034 (4)*	
C8	0.7688 (3)	0.5830 (3)	0.4799 (2)	0.0778 (7)	
H8A	0.7544	0.5131	0.5671	0.117*	
H8B	0.6986	0.6788	0.4988	0.117*	
H8C	0.8779	0.5893	0.4413	0.117*	

### Atomic displacement parameters (Å^2^)

**Table:**

	*U*^11^	*U*^22^	*U*^33^	*U*^12^	*U*^13^	*U*^23^
P1	0.0393 (3)	0.0487 (3)	0.0715 (4)	−0.0111 (2)	−0.0183 (2)	−0.0191 (2)
N1	0.0434 (8)	0.0496 (9)	0.0467 (8)	−0.0197 (7)	−0.0167 (7)	−0.0035 (7)
N2	0.0408 (9)	0.0710 (11)	0.0437 (9)	−0.0111 (8)	−0.0152 (7)	−0.0154 (8)
C1	0.0306 (9)	0.0546 (11)	0.0493 (11)	−0.0093 (8)	−0.0109 (8)	−0.0138 (8)
C2	0.0373 (10)	0.0504 (10)	0.0470 (10)	−0.0125 (8)	−0.0125 (8)	−0.0030 (8)
C3	0.0342 (9)	0.0585 (11)	0.0471 (11)	−0.0068 (8)	−0.0116 (8)	−0.0071 (9)
C4	0.0679 (14)	0.0596 (12)	0.0630 (13)	−0.0293 (11)	−0.0232 (11)	0.0004 (10)
C5	0.0784 (16)	0.0574 (13)	0.0799 (17)	−0.0289 (11)	−0.0242 (13)	0.0062 (11)
C6	0.0694 (15)	0.0636 (14)	0.0727 (17)	−0.0180 (12)	−0.0197 (13)	0.0160 (12)
C7	0.0553 (13)	0.0822 (16)	0.0469 (12)	−0.0070 (11)	−0.0174 (10)	0.0013 (11)
C9	0.0422 (10)	0.0453 (10)	0.0560 (11)	−0.0147 (8)	−0.0185 (8)	−0.0119 (8)
C10	0.0459 (11)	0.0459 (10)	0.0682 (13)	−0.0160 (8)	−0.0203 (9)	−0.0158 (9)
C11	0.0473 (11)	0.0486 (10)	0.0603 (12)	−0.0091 (8)	−0.0214 (9)	−0.0096 (9)
C12	0.0430 (10)	0.0607 (12)	0.0564 (12)	−0.0194 (9)	−0.0237 (9)	−0.0007 (9)
C13	0.0604 (13)	0.0569 (12)	0.0865 (16)	−0.0288 (10)	−0.0359 (12)	−0.0077 (11)
C14	0.0539 (12)	0.0441 (10)	0.0845 (15)	−0.0140 (9)	−0.0312 (11)	−0.0148 (10)
C15	0.0397 (10)	0.0321 (9)	0.0700 (13)	−0.0100 (7)	−0.0214 (9)	−0.0045 (8)
C16	0.0466 (11)	0.0498 (11)	0.0705 (14)	−0.0014 (9)	−0.0218 (10)	−0.0098 (10)
C17	0.0731 (16)	0.0611 (13)	0.0688 (15)	0.0051 (11)	−0.0226 (12)	−0.0104 (11)
C18	0.107 (2)	0.0487 (12)	0.0802 (17)	−0.0059 (13)	−0.0538 (17)	−0.0058 (11)
C19	0.0771 (17)	0.0552 (13)	0.102 (2)	−0.0204 (12)	−0.0572 (16)	0.0038 (12)
C20	0.0460 (11)	0.0485 (11)	0.0820 (15)	−0.0189 (9)	−0.0268 (11)	0.0018 (10)
C8	0.0765 (16)	0.111 (2)	0.0616 (14)	−0.0232 (14)	−0.0293 (12)	−0.0298 (14)

### Geometric parameters (Å, º)

**Table:**

P1—C15	1.823 (2)	C10—H10	0.9500
P1—C9	1.8304 (19)	C11—C12	1.376 (3)
P1—C1	1.8321 (19)	C11—H11	0.9500
P1—H1	1.281 (15)	C12—C13	1.374 (3)
N1—C1	1.312 (2)	C12—H12	0.9500
N1—C2	1.394 (2)	C13—C14	1.386 (3)
N2—C1	1.378 (2)	C13—H13	0.9500
N2—C3	1.381 (3)	C14—H14	0.9500
N2—C8	1.457 (3)	C15—C16	1.387 (3)
C2—C3	1.389 (3)	C15—C20	1.392 (3)
C2—C4	1.392 (3)	C16—C17	1.373 (3)
C3—C7	1.395 (3)	C16—H16	0.9500
C4—C5	1.381 (3)	C17—C18	1.386 (3)
C4—H4	0.9500	C17—H17	0.9500
C5—C6	1.377 (4)	C18—C19	1.375 (4)
C5—H5	0.9500	C18—H18	0.9500
C6—C7	1.376 (3)	C19—C20	1.373 (3)
C6—H6	0.9500	C19—H19	0.9500
C7—H7	0.9500	C20—H20	0.9500
C9—C10	1.382 (3)	C8—H8A	0.9800
C9—C14	1.390 (2)	C8—H8B	0.9800
C10—C11	1.386 (3)	C8—H8C	0.9800
			
C15—P1—C9	103.70 (8)	C12—C11—C10	120.12 (18)
C15—P1—C1	99.42 (8)	C12—C11—H11	119.9
C9—P1—C1	100.85 (8)	C10—C11—H11	119.9
C15—P1—H1	113.2 (7)	C13—C12—C11	119.64 (17)
C9—P1—H1	115.7 (7)	C13—C12—H12	120.2
C1—P1—H1	121.4 (7)	C11—C12—H12	120.2
C1—N1—C2	104.89 (16)	C12—C13—C14	120.51 (17)
C1—N2—C3	106.83 (15)	C12—C13—H13	119.7
C1—N2—C8	127.27 (19)	C14—C13—H13	119.7
C3—N2—C8	125.87 (18)	C13—C14—C9	120.28 (18)
N1—C1—N2	112.64 (17)	C13—C14—H14	119.9
N1—C1—P1	125.23 (15)	C9—C14—H14	119.9
N2—C1—P1	122.03 (14)	C16—C15—C20	118.3 (2)
C3—C2—C4	119.95 (18)	C16—C15—P1	124.29 (15)
C3—C2—N1	110.33 (16)	C20—C15—P1	117.29 (16)
C4—C2—N1	129.68 (18)	C17—C16—C15	120.7 (2)
N2—C3—C2	105.29 (16)	C17—C16—H16	119.7
N2—C3—C7	132.60 (19)	C15—C16—H16	119.7
C2—C3—C7	122.1 (2)	C16—C17—C18	120.2 (2)
C5—C4—C2	117.9 (2)	C16—C17—H17	119.9
C5—C4—H4	121.1	C18—C17—H17	119.9
C2—C4—H4	121.1	C19—C18—C17	119.7 (2)
C6—C5—C4	121.4 (2)	C19—C18—H18	120.2
C6—C5—H5	119.3	C17—C18—H18	120.2
C4—C5—H5	119.3	C20—C19—C18	120.0 (2)
C7—C6—C5	122.0 (2)	C20—C19—H19	120.0
C7—C6—H6	119.0	C18—C19—H19	120.0
C5—C6—H6	119.0	C19—C20—C15	121.0 (2)
C6—C7—C3	116.6 (2)	C19—C20—H20	119.5
C6—C7—H7	121.7	C15—C20—H20	119.5
C3—C7—H7	121.7	N2—C8—H8A	109.5
C10—C9—C14	118.61 (17)	N2—C8—H8B	109.5
C10—C9—P1	123.80 (13)	H8A—C8—H8B	109.5
C14—C9—P1	117.59 (14)	N2—C8—H8C	109.5
C9—C10—C11	120.84 (17)	H8A—C8—H8C	109.5
C9—C10—H10	119.6	H8B—C8—H8C	109.5
C11—C10—H10	119.6		
